# varCADD: large sets of standing genetic variation enable genome-wide pathogenicity prediction

**DOI:** 10.1186/s13073-025-01517-6

**Published:** 2025-08-04

**Authors:** Lusiné Nazaretyan, Philipp Rentzsch, Martin Kircher

**Affiliations:** 1 Berlin Institute of Health at Charité – Universitätsmedizin Berlin, Berlin, 10117 Germany; 2 Science for Life Laboratory, Department of Gene Technology, KTH Royal Institute of Technology, Stockholm, Sweden; 3 Institute of Human Genetics, University Medical Center Schleswig-Holstein, University of Lübeck, Lübeck, 23562 Germany

**Keywords:** Variant prioritization, Standing variation, Training data, Data biases, Variant effect prediction

## Abstract

**Background:**

Machine learning and artificial intelligence are increasingly being applied to identify phenotypically causal genetic variation. These data-driven methods require comprehensive training sets to deliver reliable results. However, large unbiased datasets for variant prioritization and effect predictions are rare as most of the available databases do not represent a broad ensemble of variant effects and are often biased towards the protein-coding genome, or even towards few well-studied genes.

**Methods:**

To overcome these issues, we propose several alternative training sets derived from subsets of human standing variation. Specifically, we use variants identified from whole-genome sequences of 71,156 individuals contained in gnomAD v3.0 and approximate the benign set with frequent standing variation and the deleterious set with rare or singleton variation. We apply the Combined Annotation Dependent Depletion framework (CADD) and train several alternative models using CADD v1.6.

**Results:**

Using the NCBI ClinVar validation set, we demonstrate that the alternative models have state-of-the-art accuracy, globally on par with deleteriousness scores of CADD v1.6 and v1.7, but also outperforming them in certain genomic regions. Being larger than conventional training datasets, including the evolutionary-derived training dataset of about 30 million variants in CADD, standing variation datasets cover a broader range of genomic regions and rare instances of the applied annotations. For example, they cover more recent evolutionary changes common in gene regulatory regions, which are more challenging to assess with conventional tools.

**Conclusions:**

Standing variation allows us to directly train state-of-the-art models for genome-wide variant prioritization or to augment evolutionary-derived variants in training. The proposed datasets have several advantages, like being substantially larger and potentially less biased. Datasets derived from standing variation represent natural allelic changes in the human genome and do not require extensive simulations and adaptations to annotations of evolutionary-derived sequence alterations used for CADD training. We provide datasets as well as trained models to the community for further development and application.

**Supplementary Information:**

The online version contains supplementary material available at 10.1186/s13073-025-01517-6.

## Background

In recent years, substantial advancements have been made in identifying phenotypically causal variants across the human genome. Machine learning-based models for prioritizing these variants have played a crucial role in this process. However, the application of machine learning on genomic data often faces challenges as comprehensive and unbiased training data is rare [1, 2]. Many collections of pathogenic and benign variants are biased towards the protein-coding genome, specifically the identification of missense (i.e., amino acid substitution) variants [3, 4]. There is no broad ascertainment of clinical variants, and few well-studied genes tend to be overrepresented in these sets [5]. Further, most of the annotated pathogenic variants have strong detrimental effects, missing out on variants with low but significant functional impact or those with complex pathways of functional impact [6–10]. Besides, datasets often contain only a limited number of variants of different molecular effects or might have mixed quality of variant interpretation [11, 12]. It is therefore challenging to train machine learning models of variant pathogenicity, especially genome-wide, across different molecular processes and effect sizes.

To overcome some of these issues, the Combined Annotation Dependent Depletion (CADD) framework [13] introduced an alternative set of proxy-deleterious and proxy-benign variants to train machine learning models for ranking all kinds of potentially disease causal variants from genome sequencing (i.e., single nucleotide, multi-nucleotide substitutions and insertion/deletion variants, as well as coding and non-coding regions of the genome). Rather than directly predicting variant pathogenicity, CADD approximates pathogenicity by modeling deleteriousness, i.e., variants that might be excluded by purifying selection during species evolution. The proxy-benign set consists of around 14 million human-lineage-derived sequence alterations which saw many generations of purifying selection and is therefore assumed to be a proxy for neutral variation. The proxy-deleterious variation is a set of simulated variants obtained by a custom genome-wide simulator of germline variation. It simulates “de novo” variants according to the substitution frequencies and insertion/deletion (InDel) lengths observed in the proxy-benign set, accommodating a local (megabase resolution) adjustment of mutation rates and asymmetric CpG-specific mutation rates. Using this data, CADD trains an unbiased learner that generalizes well to the variation in the entire human genome, outperforms other genome-wide approaches and still performs on-par or even better than highly specialized pathogenicity scores [14, 15].

Since the first publication of CADD, negative correlation has been observed between its scores and the frequency of variants from the 1000 Genomes Project [13]. This observation is in line with studies demonstrating an inverse relationship between the effect size of a variant and its frequency in the population [16–19]. Thus, the frequency of alleles with detrimental effects is controlled by purifying selection, which is the reason why this inverse relationship is skewed towards rare variants for traits strongly influenced by natural selection [20]. In contrast, variants responsible for less constrained quantitative phenotypes such as height or eye color, as well as variants underlying late-onset diseases, may be more common due to weaker selection effects [21]. This suggests that by contrasting alleles of different frequencies one can also gain insights into functional effects of variation in the human genome.

Leveraging allele frequency to gauge the functional impact of genetic variation necessitates extensive datasets of genome-wide variant data to comprehensively cover molecular effects and accurately estimate variant frequencies. When CADD was developed in 2014, variant simulations and human-species derived sequence alterations were used for its development due to the lack of large-scale genome sequencing data and variant sets. However, reduced sequencing costs have enabled comprehensive genomic datasets from thousands of individuals, facilitating the examination of the effects of variation within both coding and noncoding regions of the genome [16]. Initiatives like gnomAD [22], TOPMed [23], and ALFA [24] provide openly accessible variant data sets from thousands of individuals. While the dataset from the 1000 Genomes Project contained around 88 million variants [25], gnomAD 3.0 includes around 602 million single nucleotide variants (SNVs) and 105 million InDel variants [22]. The growing number of sequenced genomes from various initiatives is poised to enhance the interpretation of genetic variants. Larger and more broadly sampled genomic datasets not only encompass a greater abundance of rare variants but also refine our ability to accurately estimate allele frequencies between populations or geographic ancestries.

**Fig. 1:**
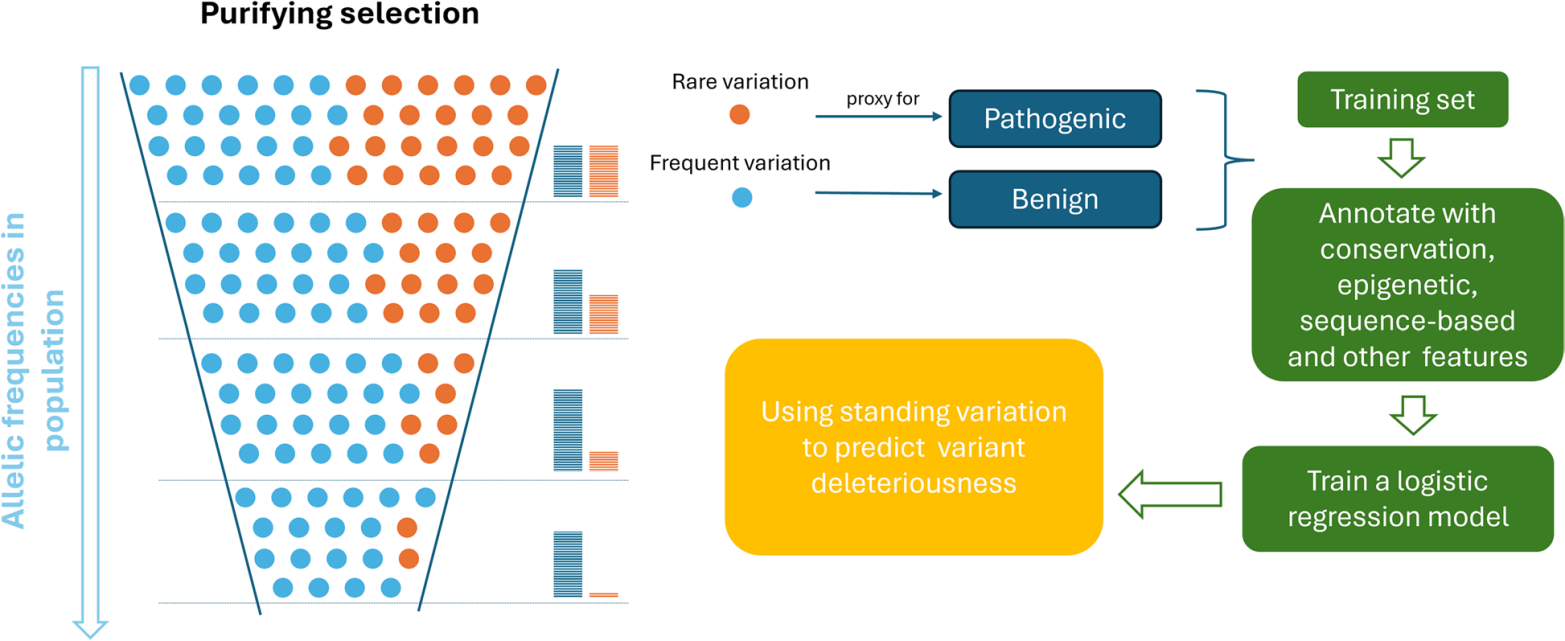
Motivation for the varCADD approach of predicting variant deleteriousness. Throughout time, pathogenic allelic changes are selected against by purifying selection, while neutral or beneficial changes can be passed along to next generations. Consequently, the frequencies of pathogenic variants are decreasing, beneficial alleles are increasing, and frequencies of neutral variants are subject to drift. For that, allele frequencies in standing variation can be used as a proxy for their deleteriousness. To train a machine learning model for variant prioritization, frequent variants from gnomAD 3.0 were used as a proxy-benign set and rare variants as a proxy-deleterious set. The resulting training set exceeds existing data sets in their size and allows for genome-wide coverage of molecular effects. The training set was annotated with sequence conservation, epigenetic, sequence-based, and other features using the CADD v1.6 framework, after which a logistic regression model was trained. The output of the model can be interpreted as a probability for a variant to have a deleterious effect on genetic function

In this manuscript, we investigate the potential of using variant data for the purpose of training genome-wide models for variant prioritization (Fig. 1). We construct several alternative training sets based on standing variation, using frequent variants as a proxy for (a higher proportion of) neutral and rare variants as a proxy for (a higher proportion of) deleterious variation. We then employ logistic regression on each of these training sets, the results of which demonstrate comparable performance with CADD. Moreover, augmenting the CADD training set of human-derived data with standing variation outperforms the original model in identifying specific types of pathogenic variants, such as stop-gain (i.e., nonsense), upstream, or 3’ UTR variants.

## Methods

### Correlation of allele frequencies and deleteriousness scores

CADD v1.6 scores for gnomAD [22] v3.0 SNVs were downloaded from the CADD webserver [26]. Their allele frequency (AF) was looked up in the gnomAD v3.0 file downloaded from the UCSC Genome Browser server [27] using tabix [28]. For each of the fifty bins of PHRED-scaled CADD scores in the interval (0, 50], 100,000 variants were selected, excluding variants with zero allele frequency (AF = 0). For PHRED scores up to 29, it was possible to extract the intended number of positions, but for variants with a PHRED score of 30 and higher, the number of available positions is decreasing, so that, for example, for the bin (49,50] only 581 variants were available. In total, 3,264,650 positions were retrieved. Data was plotted with mean allele frequency values for each of the fifty bins using Python package *seaborn v0.13.2* [29]. Additionally, the number of singletons in each bin was plotted where singletons are defined as variants with allele frequency equaling one (AF = 1) in the gnomAD v3.0 file. Spearman correlations of allele frequencies and PHRED-scaled CADD scores were measured across concatenated value pairs for all bins (*n* = 3,264,650) using *scipy v1.9.0* package [30].

### CADD training dataset

CADD training data is composed of equally large proxy-pathogenic and proxy-benign sets of SNVs and InDels. The proxy-benign set contains evolutionary derived sequence alterations (fixed or at very high frequency on the human-lineage) that saw many generations of purifying selections [13]. The proxy-pathogenic set is produced by a “germline simulator” of de novo variants that accommodates some properties of the proxy-benign variants such as substitution rates and mutations in a CpG context. Detailed information can be found in the original CADD publications [13]. The feature set of CADD v1.6 [31] contains over 100 different annotations of sequence conservation and constraint, epigenetic and regulatory activity, variant density, and others. The full list of annotations can be found in the respective CADD v1.6 release notes [32].

### gnomAD variants and dataset matching

gnomAD v3.0 variants were downloaded from the UCSC Genome Browser [26]. Sites with limited coverage (based on the available FILTER flag) or sites on alternative haplotypes, unplaced contigs, and the mitochondrial genome were dropped from analysis. As a result, we obtained 525 million SNVs and 68 million InDels that were selected according to the allele frequency thresholds defined in Table 1. Variants with an average AF > 0.5 across all individuals were not considered. For training of models based solely on standing variation, the rare and singleton variants were subsampled to match the number of variants in the frequent set in order to have balanced training data with an equal number of proxy-benign and proxy-pathogenic variants. Thereby, the substitution rates as well as the length of insertions and deletions were matched to that in the frequent set to avoid label leakage.

**Table 1:** Number of frequent, rare, and singleton variants in the gnomADdatabase as well as the human-derived set used for training of CADD v1.6. While most variants in the processed gnomAD v3.0 dataset are singletons, variants with MAF ≥ 0.001 contribute 5.9%, in absolute numbers more than the number of human-derived variants used as a benign set for training the CADD v1.6 model

Frequency	Definition	SNVs	InDels
Singletons	AC = 1	268,404,480	27,934,609
Rare	MAF < 0.001 and AC > 1	229,184,030	30,416,715
Frequent	MAF ≥ 0.001	26,255,876	8,864,604
Human-derived	Human-lineage-derived sequence alterations	14,011,297	1,688,847

For large models containing the entire set of singletons (hs-all, hfs-all), training datasets were not matched in their composition of substitution rates or length of insertions and deletions to keep the largest possible number of variants. The dataset for the hs model was sampled from the matched singleton set so that it is matched to the frequent set. Singletons for the hfs model were matched to the combined set of human-derived and frequent variants. The analysis of resulting model coefficients reveals that features containing the reference and alternative alleles or their length are of little importance for model predictions. Consequently, the exact matching seems of limited importance. All variant sets are annotated with the full set of annotations derived from CADD v1.6 [31], including variant density.

### Model training

All alternative models are trained using the CADD framework [31]. In the first step, the variant sets are annotated with all annotations used for CADD v1.6 training. In the next step, the framework imputes the annotated variants by filling missing values, binarizes categorical features, and creates feature crosses (multiplication of selected features) of select annotations. In the imputation step, annotations of variant density are omitted for models trained without those features. This results in 1028 features for the full set of annotations and 848 features without variant density annotations. The features in each dataset are normalized and used for the training of an L2-regularized logistic regression model using version 0.20.4 of *scikit-learn* [33]. In the first part of the analysis, all models are trained for 20 iterations. In the second part of the analysis, the models that consist of a combination of human-derived variants and standing variation are trained for 50 iterations. Regularization parameters remained unchanged from CADD v1.6, i.e., using an L2 penalty C = 1 and using the model after thirteen L-BFGS iterations if not otherwise stated.

### Validation sets

The validation sets used to identify the optimal iteration of logistic models are described in Additional file 1: Table S1. These are datasets previously described for CADD model evaluation [15, 34] and were chosen to cover a wide range of variant effects from pathogenicity to molecular impact. Since the datasets vary significantly in size, an unweighted average was used for model tuning (i.e., the choice of the optimal number of iterations). The optimal number of training iterations of all models based on the validation sets is printed in Additional file 1: Table S2.

The validation set used to compare trained models with CADD v1.6 and CADD v1.7 is based on the NCBI ClinVar June 2025 database release [35]. The dataset was filtered for single nucleotide variants as well as insertions and deletions of no longer than 50 base pairs mapped to the GRCh38 genome build. Variants with clinical significance “Pathogenic,” “Likely pathogenic,” “Pathogenic/Likely pathogenic,” “Likely benign,” “Benign,” and “Benign/Likely benign” were selected. Variants located on the mitochondrial genome were dropped from the analysis. The resulting dataset of 1,478,131 variants (261,148 pathogenic and 1,216,983 benign) was annotated using CADD v1.6, adding a column ‘Consequence’ based on the Ensembl VEP annotation build v95 [36]. This column was used for model comparisons on consequence level as well as for the separation of data into coding and noncoding sets (coding being defined as “STOP_GAINED,” “STOP_LOST,” “CANONICAL_SPLICE,” “NON_SYNONYMOUS,” “SYNONYMOUS,” “FRAME_SHIFT,” “INFRAME,” and noncoding being defined as “INTRONIC,” “REGULATORY,” “SPLICE_SITE,” “3PRIME_UTR,” “NONCODING_CHANGE,” “DOWNSTREAM,” “UPSTREAM,” “5PRIME_UTR,” “INTERGENIC”).

MPRA data underlying the Malinois model [37] was used for constructing a regulatory variant validation set. Variant effects were obtained from the ENCODE portal [38] for Oligo Libraries 27 (ENCFF190GIK, ENCFF244UZB, ENCFF513ZUR, ENCFF723IJU, ENCFF860FTN), 29 (ENCFF356VJF, ENCFF394RVK, ENCFF467ZQU, ENCFF660AQG, ENCFF687ONV) and 31 (ENCFF133LIK, ENCFF153VGD, ENCFF514YKU, ENCFF629BDS, ENCFF793IQS). Each of those libraries was measured in SK-N-SH, A549, GM12878, K562, and HepG2 cells. Variants with a gene regulatory effect were identified as those where the MPRA element of the variant had significant activity (A_logP and B_logP > 3), the difference between the reference and alternative element measurements was significant (Skew_logP > 4) and the absolute effect greater than or equal to 1 (|skewStat|) for at least three out of five cell types. Variants without a gene regulatory effect were defined as those where the MPRA element of the variant had significant activity (A_logP and B_logP > 4), the difference between the reference and alternative element measurements was not significant (Skew_logP < 2) and the absolute effect smaller than or equal to 0.15 (|skewStat|) for at least four out of five cell types. For the “no gene regulatory effect” variants, those annotated with splice or (non-)coding exonic effects were excluded based on Ensembl VEP v95 [36] annotations (leaving 3380 out of 3596 variants). Variants were coordinate-lifted from GRCh37 to GRCh38 using UCSC liftOver [39] and checked for strand to reorient variants for the reference allele where necessary. Ten variants were identified as shared between the experiments, but with conflicting effect labeling, and removed from both sets. A final set of 3366 neutral and 1044 variants with gene regulatory variant effect was used for benchmarking.

### Model evaluation

The models are evaluated using the Python *scikit-learn v1.1.1* package [33] (metrics: RocCurveDisplay, PrecisionRecallDisplay, roc_curve, auc, average_precision_score) and visualized with *seaborn v0.13.2* [29] and *matplotlib v3.7.0* [40]. Pearson and Spearman correlations as well as significance levels were calculated with *scipy v1.9.0* [30].

### Visualization of model coefficients

As the input data for the training of the logistic model is normalized (mean 0, standard deviation of 1), coefficients can be directly interpreted as feature importance. The 30 most important features (with the highest absolute value of the coefficient) were selected for each model and visualized in a stacked bar plot. The size of each block in a bar represents the absolute value of a model coefficient; the total length of a bar indicates the sum of coefficients. To facilitate the comparison, the features were grouped into five categories according to Additional file 1: Table S3.

## Results

### Evidence of a proxy-deleterious state of low allele frequency variants

**Fig. 2:**
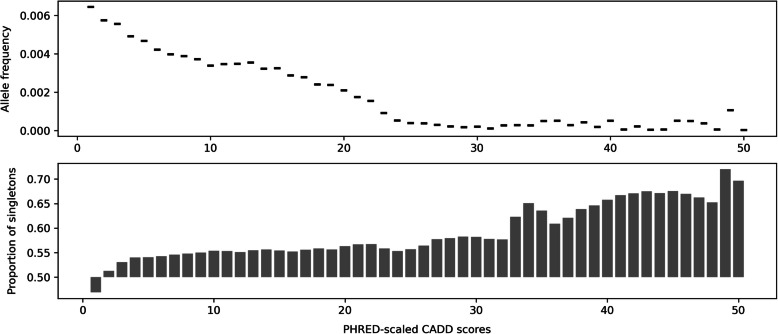
Inverse correlation between deleteriousness scores and allele frequencies. Average allele frequency for bins of PHRED-scaled CADD scores up to 50 for variants sampled from the gnomAD v3.0 database. For each bin of PHRED scores up to 100,000 variants were sampled from gnomAD v3.0. Starting from PHRED score 29, the number of available variants with the corresponding score starts to decrease sharply. For example, for 49 < PHRED ≤ 50, only 581 variants were available. At the same time, the number of singletons is increasing with PHRED score, which makes conclusions based on the right tail of the distribution less certain. In general, average allele frequency decreases with PHRED scores indicating that there is an inverse relationship between predictions of deleteriousness derived from CADD and average allele frequency

Initially, we assessed the relationship between CADD v1.6 deleteriousness scores [15] and the allele frequency for the Genome Aggregation Database (gnomAD) v3.0 [22]. This global cohort contains variants identified from genome sequencing of 71,156 individuals, excluding first or second-degree relatives, mapped to the GRCh38 genome build. In individual genomes, we had previously observed a natural depletion of variants starting at a CADD PHRED-score threshold of 20, i.e., the top 1% most extreme changes scored by CADD [13]. While this was looking at individual genomes, gnomAD is aggregating across many individuals, increasing evidence for common variants but also accumulating millions of rare and singleton variants. To analyze how these aggregated variants behave along the CADD PHRED scale, we randomly sample up to 100,000 gnomAD variants with PHRED-scaled CADD scores from 1 to 50, filtering out variants with zero allele count or allele frequency values (AC = 0 or AF = 0). Figure 2 shows the mean allele frequency for each bin of PHRED scores as well as the proportion of singletons in each bin. We observe an inverse relationship between the average allele frequency across the variants in a bin and PHRED scores, i.e., the predicted deleteriousness of a variant in the gnomAD set is negatively correlated with its frequency. This relation is especially clear for the lower-scoring and largest part of the genome, i.e., average allele frequency decreasing up to PHRED 25 (with scores above 25 representing less than 0.3% of the genome), and plateaus for higher deleterious scores with some variation due to limited observations. Considering variants across all PHRED bins, scaled CADD scores and allele frequencies show a Spearman correlation with allele frequencies of − 0.116 (*n* = 3,264,650, *p*-value < 1.171 × 10^−157^) or − 0.131 (*n* = 1,443,979, *p*-value < 1.13 × 10^−187^) when excluding singletons. The lower panel of Fig. 2 illustrates that the proportion of singleton variants is increasing with deleteriousness measured by CADD from about 55% at PHRED 20 to about 70% at PHRED 50, further highlighting that the average allele frequency measured in the top panel is dominated by singletons.

Studies using genome and exome sequencing data allow quantitative assessment of purifying selection. For example, Dukler et al. [41] estimate that ~ 0.4–0.7% of the human genome is ultra-selected, which suggests 0.26–0.51 strongly deleterious de novo mutations per generation. Moreover, singletons, variants observed only once in the entire cohort and potentially harboring de novo variation, are more likely to be located in functionally important loci [42]. However, new mutations are challenging to assess as they are hard to detect and have fitness effects that are difficult to measure [41]. Nonetheless, various studies demonstrate that singletons, ultra-rare and rare variants accumulate in certain disease cohorts [18, 43–47]. This evidence along with the highly significant correlation of CADD scores with allele frequencies supports the idea that information about allele frequency can be leveraged further, delivering new biological insights and improving variant prioritization.

### Composition of alternative datasets

Capitalizing on the idea of purifying selection, we want to use variation of different allelic frequencies as datasets for training machine learning models for variant prioritization. For that, we continue with gnomAD v3.0. Due to the limited or questionable coverage of downstream annotations used for alternative haplotypes, unplaced contigs and the mitochondrial genome, we drop these variants from further analysis. As a result, we obtained 525 million SNVs and 68 million InDels. Much of the variation considered (51.1% of SNVs and 41.3% of InDels) has an allele count of 1 (AC = 1, singleton) in the database, i.e., it is observed only in a single individual, on one of their haplotypes. These singleton variants could potentially constitute the largest group of a proxy-pathogenic variant set for model training.

Further, we define all variants below a minor allele frequency (MAF) of 0.1% as rare variants and regard them as a second potential proxy-pathogenic set (MAF < 0.1% & AC > 1). Thus, as a proxy-benign set, we define frequent variants, i.e., those greater than 0.1%. Although there is no universal definition of how often a variant should be observed in a population to be called frequent, numbers between 0.1% and 1% have been used before. They are considered to be conservative frequency cutoffs for recessive (1%) and dominant (0.1%) diseases, respectively [48]. Further, population genetic studies demonstrate that severe disease-causing variants have much lower frequency than these thresholds [49]. Thus, a cutoff of 0.1% for the proxy-benign set seems to deliver a reasonable separation from proxy-pathogenic variants for our purpose.

As the number of all variants differs significantly throughout the groups, with “singletons” being the most abundant and “frequent” variants the least abundant, we sample all the “singleton” and “rare” variants to match the number of frequent variants, so that each potential training set contains 26,255,876 SNVs and 8,864,604 InDels (Table 1). We use both “singleton” and “rare” variant sets as proxy for deleterious variation and “frequent” variants as proxy for benign variation.

### Features of alternative models

The CADD v1.6 training dataset is composed of 1028 features [15]. Among them are the reference and the alternative allele, as well as the inferred transition/transversion state. We have observed that the substitution rates of the four nucleotide bases adenine (A), guanine (G), cytosine (C) and thymine (T) differ across the three allele frequency groups defined above. For example, the proportion of C-to-T substitution in the set of “frequent” variants is 40.1%, whereas in the “singleton” set it is only 29.8% (Additional file 1: Fig. S1). These shifts in representation of reference and alternative alleles as well as transition/transversion class in these alternative training sets can potentially cause label leakage and misguide model training. As a result, a model might for example assign a lower deleteriousness score to a variant with C to G substitution without any biological reasoning, but only because it is more frequent in the proxy-benign training set than in the proxy-deleterious training set. Therefore, we matched the substitution rates across the training sets to those of the “frequent” set.

Another set of features used to train CADD is derived from variant density. This set constitutes a total of 180 features, e.g., distance or number of rare, frequent, or singleton SNV in a certain window in the BRAVO variant database [23] as well as combinations of these features with selected annotations from the feature set (see Section *Model training* in *Methods*). BRAVO and gnomAD variant frequencies as well as presence/absence states will for obvious reasons be correlated, rendering these annotations potentially harmful for an unbiased model training (due to another potential source of label leakage). Hence, we train all models including and excluding annotations of variant density and compare their results. Otherwise, we use all features of CADD v1.6 to train a logistic regression model for each of the dataset pairs listed in Table 2. Additionally, we train a model on the original CADD dataset (human-derived versus simulation) excluding features derived from variant density annotations to assess the impact of these annotations on models not based on standing variation.

**Table 2:** Datasets used for training CADD v1.6 and our alternative models. Each pair of datasets is trained twice, including and excluding the annotations of variant density, to assess how those influence model predictions. Additionally, a model is trained based on the original CADD training set (human-derived vs. simulated) excluding variant density features

Identifier	Proxy-benign	Proxy-deleterious	Variant density
fr	Frequent	Rare	Excluded
fr’	Frequent	Rare	Included
fs	Frequent	Singleton	Excluded
fs’	Frequent	Singleton	Included
rs	Rare	Singleton	Excluded
rs’	Rare	Singleton	Included
v1-6	Human-derived	Simulated	Excluded
v1-6' (CADD v1.6)	Human-derived	Simulated	Included

### Training optimized alternative models

In terms of machine learning, it is important to understand two specific characteristics of training CADD models. For one, CADD trains a surrogate model, i.e., its score assesses the putative deleteriousness of genomic variants, but it is applied to predict a wide range of variant effects, including pathogenicity, molecular function, and variant impact. Furthermore, the training set of CADD has a substantial degree of mislabeling. For example, the set of simulated variants contains a high number of neutral variants, as simulated variants are “randomly” sampled across the genome, including regions of no functional constraint [15]. On the other hand, the proxy-benign set will contain functional changes that underlie human-derived traits. For that, tuning and evaluation of CADD-like models are not sufficient on the holdout set of the training data but need external validation sets that cover various variant effects and are less prone to mislabeling.

Models for all current CADD releases (v1.4–1.7) have been trained using bulk logistic regression with 20 iterations. After each iteration, the model performance on external validation sets has been assessed, and the 13th model has been selected as the one with the best generalization [34]. To optimize alternative models, we follow the same scheme of training for 20 consecutive iterations. Due to the change in training data, we studied the effect on optimal training duration as well. For that, we assess and plot the models’ performance using Area Under the ROC (AUROC) curve and Area Under the Precision Recall Curve (AUPRC) after each iteration on a combination of 10 validation sets (see Section *Validation sets* in *Methods*). Based on the results, we identify the best iteration for each of the trained models (Additional file 1: Fig. S2) and use them for all subsequent analyses. The selected iterations can be found in Additional File 1: Table S2.

### State-of-the art models can be trained from standing variation

In the first step of the analysis, we want to assess whether the alternative models with labels based on standing variation can achieve state-of-the-art performance for variant prioritization. For that, we compare the AUROC and AUPRC of newly trained models with that of CADD v1.6 and newly released CADD v1.7 [34] on an independent validation set created from NCBI ClinVar data (not used for model tuning). To create the dataset (see Methods section *Model evaluation* for details), we obtained the most recent ClinVar release and filtered SNVs and InDels with GRCh38 coordinates for their clinical significance benign, likely benign, pathogenic, and likely pathogenic. Further, we limited their length to a maximum of 50 base pairs. The resulting dataset contains 1,478,131 variants, 261,148 of which are pathogenic and 1,216,983 are benign.

**Fig. 3:**
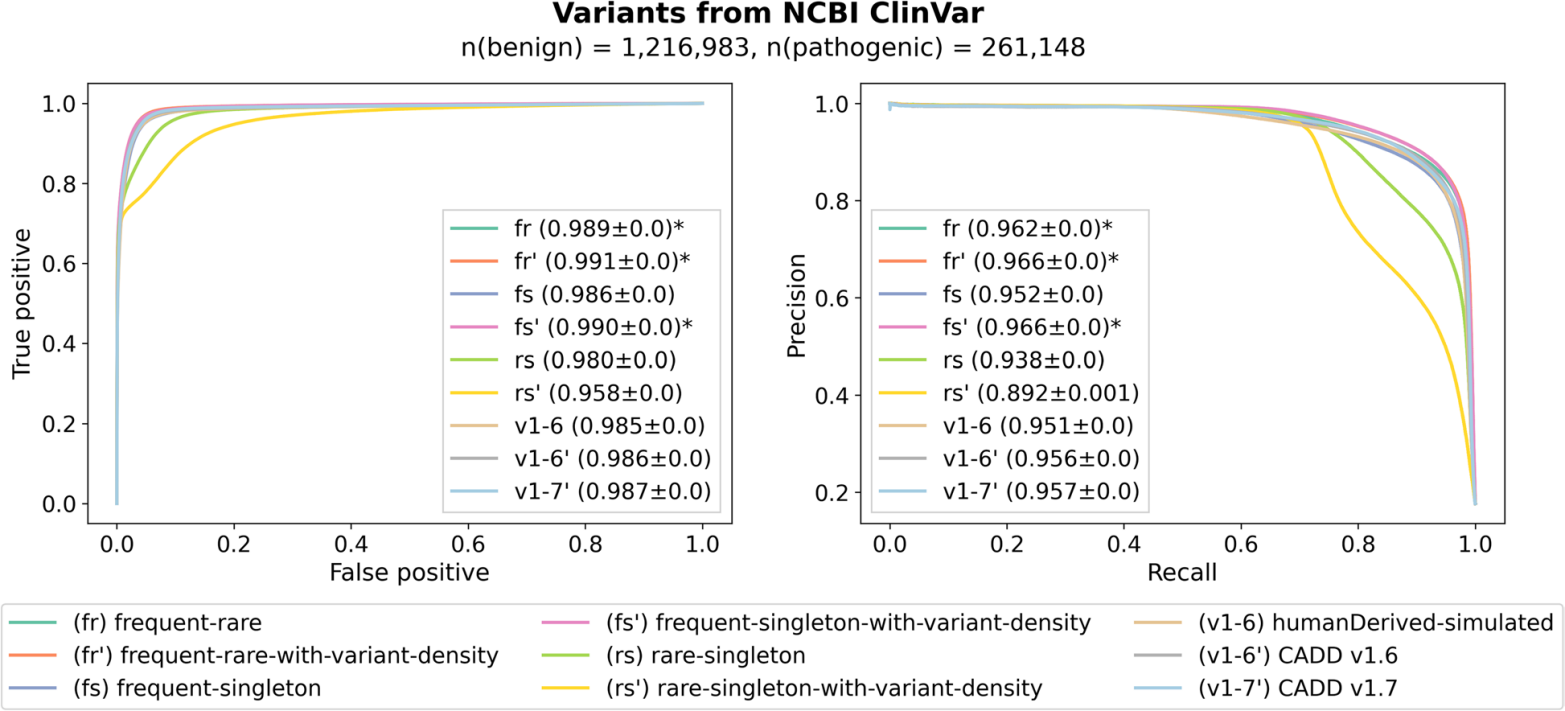
AUROC and AUPRC of trained models compared with CADD v1.6 and v1.7 on a validation set derived from NCBI ClinVar. Asterisks mark the models with the highest scores. The worst performing models are based on the rare vs. singleton datasets which might be explained by the fact that these proxies for pathogenic and benign sets are very close and thus more difficult to discriminate. The rest of the models have similar performance to CADD v1.6 and CADD v1.7 and, hence, can be used to predict pathogenicity of genetic variation. Models that include variant density annotations are indicated with an apostrophe

Figure 3 shows the ROC and PRC of trained models on the NCBI ClinVar validation set. We observe that all models achieve a high classification rate, with the worst performing model in terms of recall having an AUROC of 0.958 (rs’, rare-singleton-with-variant-density) and the best performing model of 0.991 (fr’, frequent-rare-with-variant-density). For precision scores, differences between models are rather small in most of the comparisons. The best alternative models are fr’ and frequent-singleton-with-variant-density (fs’), both slightly better than CADD v1.6 (0.966 vs. 0.956) and CADD v1.7 (0.957). The worst models are rare-singleton with and without variant density annotations (rs’ and rs). We speculate that the composition of such training sets is not discriminating between variant effects enough, so that a model has more difficulties distinguishing between true pathogenic and benign variants properly. Across all models, performance differences were too small to derive a clear conclusion in favor of or against one of the models. However, we note that alternative models based on frequent-rare and frequent-singleton sets show comparable performance to CADD v1.6 and CADD v1.7, despite the more comprehensive feature set of v1.7 (discussed below).

Further, the inclusion of variant density annotations seems to have a mixed effect: for models based on frequent-rare and frequent-singleton datasets, the performance seems to improve slightly; for models based on the rare-singleton set, the opposite is the case. It is likely that annotations of variant density are beneficial for training sets with higher discriminative potential between proxy-deleterious and proxy-benign sets because they can be used to distinguish these variants further, whereas in training data with higher label noise, these additional features and their noise hinder model training. Importantly, we do not observe a substantial label leakage due to these annotations as the performance of frequent-rare and frequent-singleton models on the external ClinVar validation set does not deteriorate with their inclusion.

### Models have similar discriminative power for highly deleterious variants

**Fig. 4:**
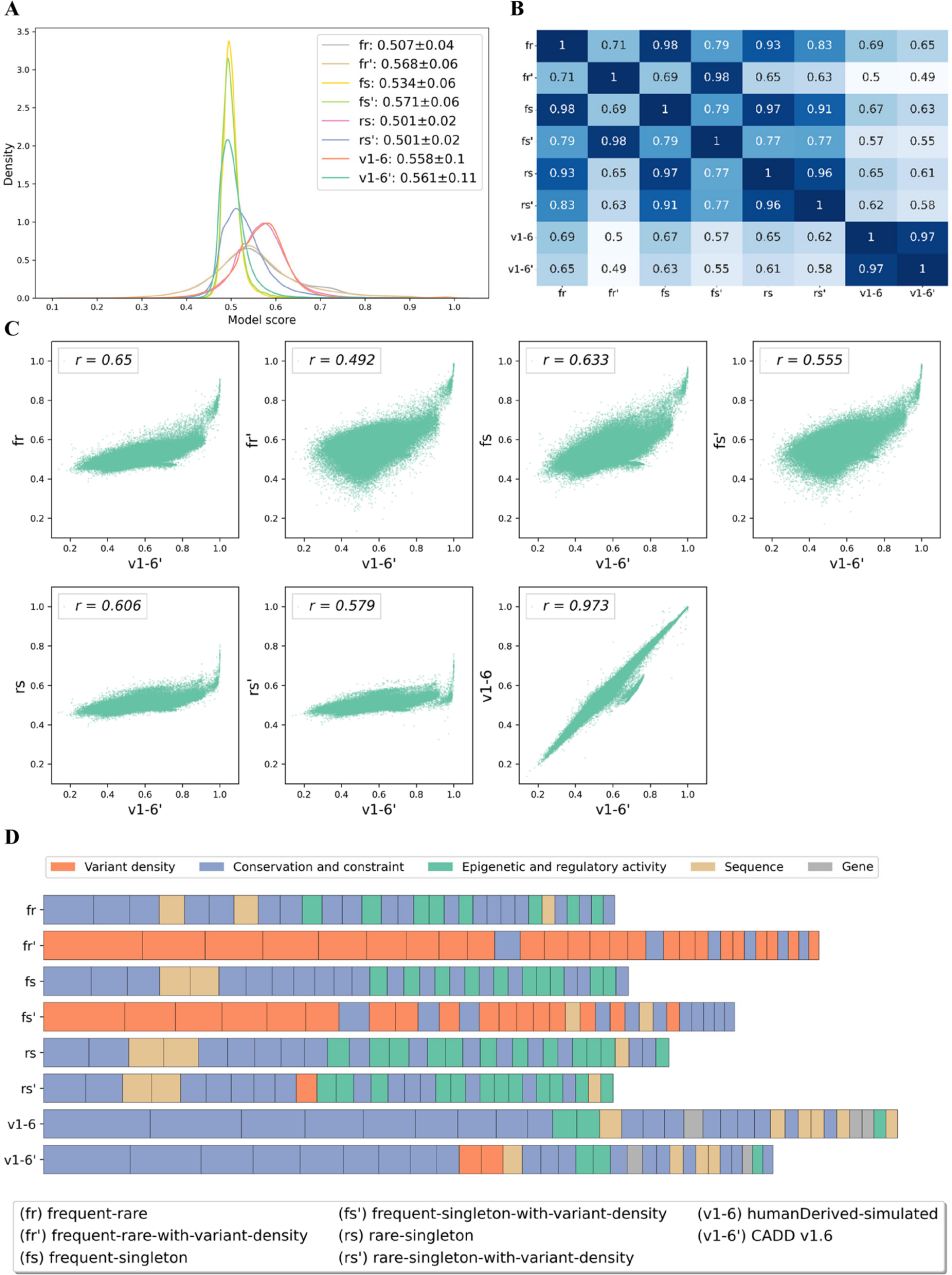
Distribution and correlation of scores as well as model coefficients of trained models. **A**–**C** are based on a set of 1 million randomly sampled potential SNVs from the reference genome. Model labels are used as defined in Table 2. The label v1-6' refers to the published CADD model; v1-6 is a model trained on the original CADD v1.6 data but without the annotations of variant density. **A) **Distribution of model scores. Models based on sets of frequent-rare and rare-singleton variants do not exhibit major changes in the score distribution with the inclusion of annotations of variant density. The model based on the set of frequent-singleton variants seems to be more sensitive to additional annotations. **B **Pearson correlation of model predictions. Models fr(frequent-rare-with-variant-density) and fs(frequent-singleton-with-variant-density) are highly correlated with each other (Pearson rho = 0.98) and less correlated with their respective versions without annotations of variant density (fr: 0.71, fs: 0.79). **C **Scatter plots of model predictions (y-axis) and CADD v1.6 scores (x-axis). Higher scores indicate higher predicted deleteriousness. Models tend to agree on highly scoring variants but diverge in scoring less deleterious variants. **D **Model coefficients of the first 30 most important features or feature combinations. The features are color-grouped to facilitate interpretation. The size of each block in a bar represents the absolute value of a model coefficient; the total length of a bar indicates the sum of coefficients. Conservation and constraint features are of high importance for almost all models except for those based on frequent-rare and frequent-singleton sets trained with the full set of features (fr’ and fs’). For those, annotations of variant density dominate the model predictions

While the overall performance seems similar across models, we wanted to understand whether there is a notable difference between model scores (Fig. 4). We started our analysis by taking a closer look at a histogram/kernel density distribution of scores produced by different models calculated on predictions of 1 million potential SNVs randomly sampled from the human genome (Fig. 4A). Here, two different distribution shapes can be distinguished. Models like rs (rare-singleton), rs’ (rare-singleton-with-variant-density) and fr (frequent-rare) have narrow-shaped model score distributions (i.e., less variance) compared to all other models. This indicates that they do not exploit the entire score range, therefore, having lower resolution not only on the tails of the score distribution but also in the middle range. Further, the resulting deleteriousness scores of these models are highly correlated (Fig. 4B). Models trained with variant density features tend to have higher average scores and higher variance. This indicates that annotations of variant density have effects on model outcomes, shifting the average predicted deleteriousness to a higher (fr and fs) or lower value (rs and v1-6).

Next, we analyzed the correlation of alternative models with CADD v1.6 scores that are based on the same model features as these new models. Figure 4C shows pairwise scatterplots of all models with CADD v1.6 scores for the 1 million SNVs selected above. A higher score indicates a higher predicted probability for a variant to be deleterious. While the CADD model trained without annotations of variant density (v1-6) is highly correlated (Pearson correlation coefficient of 0.97), the models trained on standing variation are only moderately correlated with CADD v1.6 scores (Pearson correlation coefficients from 0.49 to 0.65). The highest correlation is with the fr’ (frequent-rare-with-variant-density) and the lowest correlation is with fr. Hence, in the frequent-rare and frequent-singleton training sets, annotations of variant density seem to play an important role for score ranks. Importantly, most of the models tend to agree on the score of highly deleterious variants (according to CADD v1.6) but have different predictions when the functional impact of a variant might be more ambiguous. This is a known trend in scoring of variant deleteriousness, as for example pathogenic variants have a significant molecular function impact that is easier to recognize. On the contrary, weaker or more quantitative effects are more difficult to capture and model, leading to disagreement between model predictions. This might also explain the only moderate correlations between models, as in the sampled set of 1 million variants, the proportion of strongly deleterious variants is expected to be low, whereas variants with no or weak effects are expected to be most abundant.

### Alternative models do not overfit on variant density information

To further explore the differences in model predictions, we examine the coefficients of trained models as they can give an insight into the underlying causes of different score distributions and correlations. Figure 4D shows the assigned feature groups of the first 30 most important features in each model (Additional file 1: Table S3). As feature values are normalized for all these models, coefficients can be compared directly.

In alternative models with variant density features, those features seem dominant for model decisions. An exception here is the model rs’ (random-singleton-with-variant-density) where annotations of variant density have lower coefficients and are less frequent in the top 30 features. This observation supports the suspicion that singleton and rare variants are rather similar in their relation to variant density, having only minor differences in the annotations of variant density, so that model training cannot use them to separate the positive and the negative set. Contrary, for fr’ (frequent-rare-with-variant-density), 24 out of 30 features and, for fs’ (frequent-singleton-with-variant-density), 19 out of 30 most important features belong to the group of variant density, having also the highest coefficients. Hence, a bigger proportion of the variance explained by the model is attributed to the annotations of variant density.

Interestingly, in humanDerived-simulated, i.e., the original CADD models, conservation and constraint annotations are very dominant among the most important features (Fig. 4D), whereas in models based on standing variation, annotations of epigenetic and regulatory activity as well as sequence-based features are of considerable importance. This might be explained by the consideration that standing variation contains more recent allelic changes with fewer generations of purifying selection as the evolutionary variants, and hence, are less well separated by species conservation information. Consequently, the models trained on standing variation would put less weight on these features to distinguish between the proxy-pathogenic and proxy-benign variants in the training data.

### Substituting simulated variants for singleton variation in CADD

One of the drawbacks of CADD is its proxy-pathogenic training set based on a simplified simulation of de novo variants that might not reflect the entire complexity of natural selection and not perfectly reproduce the true distribution of variants across functional elements [50]. For that, replacing the simulated data with real de novo variants might be advantageous for deleteriousness scoring. De novo variants are identified using trio-sequencing, as each human carries up to about 80 de novo variants [51–53]. To replace the CADD dataset of 14 million simulated SNVs, one would need around 175,000 trios. However, trio studies are often elaborate and expensive, so that even with the largest initiatives of trio sequencing like the Simons Powering Autism Research (SPARK, 50,000 trios) [54, 55] or the Deciphering Developmental Disorders (DDD, 14,000 trios) study [54, 55], the amount of available data is not (yet) sufficient to replace the proxy-pathogenic set of CADD. Singletons can serve as an approximation for de novo variants as these rare variants also contain many newly occurring variants that have seen little purifying selection. For that reason, we replace the simulated data in the original CADD training set with the singleton set derived from the gnomAD v3.0 database and train another CADD-like model.

As the singleton set contains a much higher number of variants than the human-derived set (Table 1), we train balanced (hs for humanDerived-singleton) and unbalanced (hs-all for humanDerived-singleton-all) models (Table 3). For the balanced model, a subsample of the singleton set is used (14,011,297 SNVs and 1,688,847 InDels), while all singleton variants (268,404,480 SNVs and 27,934,609 InDels) are contrasted with the human-derived set for the unbalanced model, where we increase the class weights of the human-derived variants respectively. We speculate that the higher number of training variants could lead to a better coverage of rare annotations as well as broader genomic regions, which might be beneficial for the generalization power of the final model. The models are trained for up to 50 iterations, as we suspected that large datasets might require more intensive training to achieve the best results. As with previous models, the final number of iterations is chosen based on validation sets (see Methods section *Training optimized alternative models*, Additional file 1: Fig. S3).

**Table 3:** Overview of models with a combined set of training variants. For each combination, a balanced (with a sample of singleton variants) and an unbalanced (with the entire set of singleton variants) model was trained

Identifier	Proxy-benign	Proxy-deleterious	Balanced
hs	humanDerived	Singleton	Yes
hs-all	humanDerived	Singleton	No
hfs	humanDerived + frequent	Singleton	Yes
hfs-all	humanDerived + frequent	Singleton	No

**Fig. 5:**
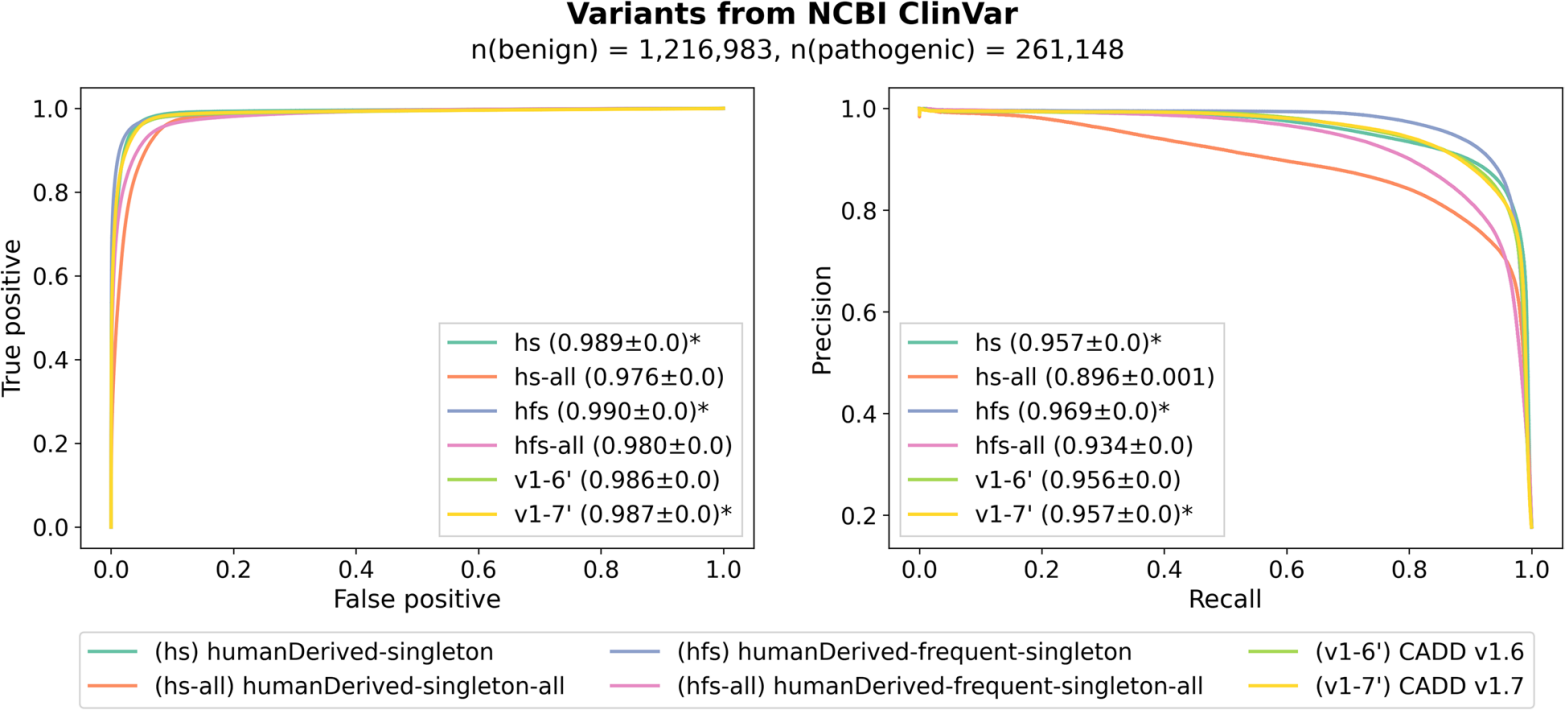
Performance of the alternative models trained on the combined sets of human-derived variants and standing variation. The left panel shows the ROC, and the right the precision-recall curve. Asterisks mark the models with the highest scores. While the models trained on balanced datasets have comparable performance, the ones trained with the entire set of singleton variants perform worse, especially for precision-recall. The model trained on the augmented dataset (hfs), i.e., on a combination of the human-derived variants and the frequent variation as a proxy-benign set, and the singleton variants as a proxy-deleterious set, performs best compared to other alternative models as well as CADD v1.6 and CADD v1.7

Figure 5 shows the results on the ClinVar validation set. In terms of recall of pathogenic variants, the model hs (humanDerived-singleton) and hs-all (humanDerived-singleton-all) perform similarly (AUROC 0.989 vs. 0.976), with the smaller training set resulting in the better model. Contrary to our expectations, and despite accounting for class imbalance during the training of the logistic regression model, there is no improvement with the larger dataset containing all singleton variants, and the precision-recall score is notably lower (0.896) than that of the balanced model (0.957). We note that the hs model has a slightly higher AUROC than CADD v1.6 (0.989 vs. 0.986) and CADD v1.7 (0.987). Conceivably, the large set of singletons introduces a significant amount of noise into the training data that outweighs the potential advantages of having larger training data.

### Augmenting human-derived variants with standing variation increases training data and improves performance

Large datasets of human variation may not only improve the original proxy-pathogenic set of CADD but may also allow for an extended proxy-benign set. The proxy-benign set of human-lineage derived fixed or high-frequency variants is based on variants that saw many generations of purifying selection, while being fixed among all humans or substantially increasing in frequency in humans. The purifying selection probably goes along with substantial shifts in functional constraint measures, like species conservation. Using these measures in distinguishing high and low effect variants might be advantageous in many cases; it may however miss effects of recent evolutionary changes that might also have an impact on fitness [56]. The analysis of model coefficients in the previous section showed that conservation and constraint features are less important when training models with standing variation; hence, including standing variation in the training set might be beneficial in terms of accounting for recent evolutionary changes. Thus, we train an additional model with an augmented set of proxy-benign variants that contains a combination of human-derived and frequent variants and contrasts it with a set of singleton variants.

Again, we train balanced (hfs for humanDerived-frequent-singleton) and unbalanced (hfs-all for humanDerived-frequent-singleton-all) models (Table 3). For the balanced model, a subsample of the singleton set is used (40,267,173 SNVs and 9,033,4893 InDels). For the unbalanced one, all singleton variants (268,404,480 SNVs and 27,934,609 InDels) are contrasted with the combined proxy-benign set. Note that all sequence differences in the original CADD proxy-benign training set were checked against the 1000 Genome database to exclude variants variable in the human population (requiring an allele frequency of more than 95%) (Kircher et al., 2014). As described before, models are trained for 50 iterations and compared using the ClinVar validation set.

Figure 5 shows the performance of models compared with CADD v1.6 and CADD v1.7. In terms of recall of pathogenic variants, we observe a slight improvement for the balanced model (hfs, 0.990) compared with CADD v1.6 (0.986) or v1.7 (0.987) but deterioration for the unbalanced one (hfs-all, 0.980). The increased performance of the hfs model is more pronounced when looking at precision-recall (0.969 vs. 0.956 and 0.957 for CADD v1.6 and v1.7, respectively), but so is the deterioration of the unbalanced model hfs-all (0.934), presumably due to the introduction of noise from the very large set of singleton variants. We conclude that the inclusion of frequent standing variation improves the deleteriousness prediction of CADD scores.

We repeated the analysis of feature importances for the hs and hfs models (Additional file 1: Fig. S4), noticing that annotations of variant density play a significant role for these models but to a lesser extent than for models based solely on standing variation (Fig. 4D). Instead, species conservation and other constraint measures gain importance here, which we attribute to the presence of the human-derived variants in these training sets.

### Alternative models outperform in various variant groups

ClinVar-based validation datasets seem somewhat saturated, i.e., the performance of most models is so high that selecting one best model is statistically feasible, but potentially not meaningful. Thus, we tried to assess model performance on ClinVar by variant consequences, anticipating clearer differences between models for the more difficult-to-differentiate functional consequence levels. In this analysis, we include the previous models trained solely on standing variation, excluding those based on the rare-singleton set as they had the lowest performance across all consequence levels.

**Fig. 6:**
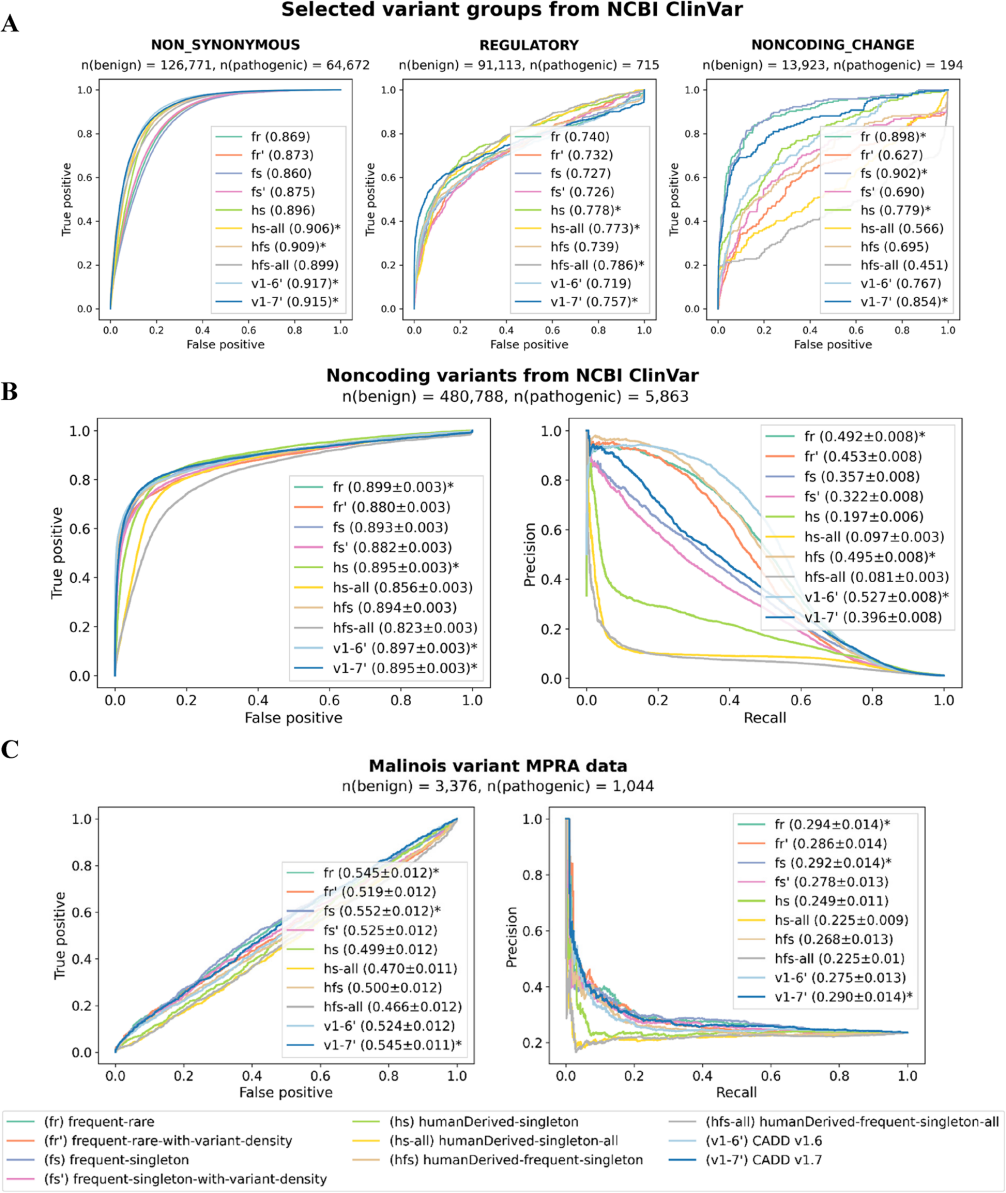
Model performance in various variant groups as well as on noncoding variants from NCBI ClinVar and experimentally measured gene regulatory variant effects. Asterisks mark the models with the highest scores. **A** Performance for selected groups of predicted molecular effects in NCBI ClinVar. Neither alternative models nor CADD v1.6 and v1.7 outperform other models in a majority of variant groups. Instead, several models have the highest recall rates in one or a few consequence labels (see Additional file 1: Fig. S5 and S6 for a full comparison). For example, CADD v1.6 has the highest recall for nonsynonymous, hfs-all (humanDerived—frequent-singleton) for regulatory and CADD v1.7 for 3’UTRs variants. **B **Model performance aggregated over NCBI ClinVar noncoding variants. Differences between models are clearer when comparing them using a set of noncoding variants from the NCBI ClinVar database. While most of the models have comparable performance in terms of recall, a higher precision is achieved by the hfs (humanDerived-frequent-singleton) and frequent-rare models. **C **Model performance on gene regulatory variants identified from experimental assays. Massively Parallel Reporter Assay (MPRA) data underlying the Malinois model [37] was used for constructing a regulatory variant validation set. A set of 3366 neutral and 1044 variants with gene regulatory variant effects across multiple human cell lines were identified from this data. All models are challenged by this data set with a close to random performance based on AUROC. VarCADD models fr and fs as well as CADD v1.7 show the best performance on this data set

The evaluation of models delivers mixed results as there is not one model that outperforms all others across all or most variant groups (Additional file 1: Fig. S5 and S6,Fig. 6A). For example, CADD v1.6 is better than alternative models in AUROC of nonsynonymous (0.917) and non-canonical splice-site variants (0.973); whereas, for example, the humanDerived-frequent-singleton-all model (hfs-all) ranks first for regulatory (0.786) and stop-gain variants (0.730). The frequent-singleton (fs) model ranks first for variants causing changes in noncoding exons (0.902). Most of the differences are small though (average standard deviation of AUROC values across consequence labels of 0.041), with models generally performing well for non-canonical splice variants, nonsynonymous amino acid exchanges, and downstream gene variants in ClinVar, and models generally lacking in performance for stop-gain, frameshift, and synonymous variants in ClinVar. We note that this will not reflect the general ability of current models in predicting these functional effects but is likely influenced by ClinVar’s ascertainment effects of variants with these consequence labels. Accounting for the AUROC performance across the 15 consequence labels (Additional file 1: Fig. S5 and S6), CADD v1.7 shows the best performance, followed by the hfs model and then by CADD v1.6 as well as the fr model. The improved CADD v1.7 performance is most likely due to a substantially updated feature set, which does not allow for an equal comparison here. For example, CADD v1.7 includes updates to gene model annotations, species conservation tracks, and other genome-wide constraint measures, as well as the integration of sequence-based models for protein-coding sequence alterations and gene regulatory sequence effects. However, any performance of varCADD models that exceeds the performance of CADD v1.7 highlights the potential for improved CADD models from the incorporation of such larger labeled training sets.

In the next step, we analyze the validation set aggregated over coding (Additional file 1: Fig. S7) and noncoding variants (Fig. 6B) (for the definition of coding and noncoding regions, see the Methods section on *Model evaluation*). While hfs, f, and fsmodels show the best performance for coding variants (Additional file 1: Fig. S7), which make up most of the validation dataset with over 67% of all and 97% of the pathogenic variants, differences between models are small and probably not meaningful. The most notable differences between models can be observed for noncoding variants. Again, the large models containing all gnomAD singleton variants have lower AUROC scores for predictions of noncoding variants (hs-all, 0.85; hfs-all, 0.823) and significantly lower AUPRC scores (hs-all, 0.097; hfs-all, 0.081). These models seem to assign higher deleteriousness to ClinVar variants, although their mean score on the 1 million variants is in general lower than that of other models (Additional file 1: Table S4). Most of the alternative varCADD models have significantly lower precision-recall scores on noncoding variants compared to CADD v1.6 (0.527). Exceptions are the model based on the combination of human-derived variants and standing variation (hfs, 0.495) and the frequent-rare model (fr). Thus, the hfs model can be highlighted for both coding and noncoding effects, suggesting a benefit from augmentation of the CADD human-derived variant dataset with frequent and singleton standing variation.

With non-coding sequence effects being a major challenge for the prioritization of variant effects, gene regulatory effects in promoter and enhancer regions have been a focus of research over the last decade. We have previously benchmarked variant effect models on gene regulatory variants measured with so-called saturation mutagenesis massively parallel reporter assay (satMutMPRA) experiments [34, 57]. Such data test variant effects independent of their presence in population data and densely across well-defined regulatory elements. However, the disadvantage of satMutMPRA data is the limited representation of different loci across the genome. Recently, other labs have obtained comprehensive variant measurements for population variants across the genome using reporter assays in multiple cell types [37]. Thus, we are evaluating the model performance on variants identified with and without significant effects across cell types (see Methods section on *Validation sets* for details). Figure 6C shows ROC and precision-recall performance across various models. We find that CADD v1.7 with its updated feature set is on par with the fr and fs models developed from human standing variation, while hs and hfs models show lower performance for this task. This again highlights the potential of achieving similar or better performance from an alternative labeled training data set rather than only relying on updates and advances in model features, suggesting that the combination of both may produce even better results.

## Discussion

The prioritization of disease-causing variants with machine learning methods is of increasing importance for clinical genetics and precision medicine, making comprehensive and reliable genomic training data essential. Here, we presented several training sets based on human standing genetic variation and showed that they are competitive with CADD v1.6 or v1.7 state-of-the-art models for genome-wide variant prioritization that are based on evolutionary derived sequence changes and de novo variant simulations.

The idea of using human genetic variation was motivated by the observation that deleteriousness scores like CADD v1.6 are correlated with the observed allele frequency of variants in the population. This observation is in line with the rare disease model that argues that Mendelian diseases are caused by individually rare alleles of large effects and is supported by evolutionary theory which predicts that disease-causing variants are linked to reduced fitness and are selected against, so their frequency in the population should be low [58–60]. At the same time, the presence of pathogenic variation in the human genome is explained by detrimental variants not being completely eliminated by purifying selection as mutations are being constantly created in a large population, some diseases only causing modest effects on fitness, or late-onset diseases and recessive effects with only limited purifying selection [61]. These considerations support the idea that leveraging variation in the human population can be beneficial for predicting deleteriousness.

While the application for genome-wide prediction of variant deleteriousness is novel, several methods have pioneered the integration of genetic variation data together with functional genomics for deriving measures of sequence constraint. For example, LINSIGHT integrates information on genetic polymorphisms within the human genome and its divergence from closely related species to assign a likelihood to noncoding genomic segments to have an impact on fitness [62]. It relies on the idea that signals of past natural selection offer valuable insights into today’s phenotypic importance. Another evolutionary based tool, PrimateAI, builds a deep neural network for pathogenicity assessment of missense variation using variants from gnomAD with an allele frequency of > 0.1% [5]. The authors expanded a rather small set of common human missense variants with an extensive set of frequent missense variation observed in primates, showing that variants commonly observed in other primates are largely benign in humans. In this study, we build upon these previous observations to build genome-wide ML models of variant effects, showing that common human variation can be used as a proxy for benign allelic changes in all genomic regions, whereas (ultra-)rare variation can approximate deleterious variation in a binary classification approach. We show that this approach can compete with state-of-the-art methods for variant prioritization like CADD v1.6 and v1.7 and overcomes some limitations of its training data, like the artificial nature of simulated variants and a masking of human-derived changes in the calculation of sequence constraint measures.

Leveraging standing variation accounts for more recent evolutionary changes in the human genome, that are especially common in regulatory elements and that tend to be less conserved than, for example, protein-coding DNA [63, 64]. Our results show that models based on standing variation put less focus on constraint and conservation measures during the model training and better utilize epigenetic and regulatory activity for the assessment of functional impact of variation. Standing variation offers a reasonable alternative for the simulated proxy-deleterious data as it has a natural representation of variants across the functional elements compared to the simulated data, which only adjusts mutation rates in large genome windows. Further, datasets based on standing variation are already large, allowing a comprehensive sampling of feature values and in principle also allow for more complex models. The coverage of rare annotations and broad sampling of genetic regions is instrumental for better modeling of the underlying biological processes in general and an improved variant prioritization in particular. The size of these datasets will continue to increase with an increasing number of sequenced genomes, improving the quality and accuracy of estimated allelic frequencies and enabling regression rather than classification methods.

The human-derived data used by CADD and other tools [2, 5, 65] offers a large dataset of likely benign genetic changes that, however, needs elaborate curation of features and limits the choice of accessible annotations. For example, the conservation scores PhyloP and PhastCons must be recalculated for CADD, excluding the human reference sequence. This is necessary because human-derived variants would be represented in the human reference sequence and, consequently, these alignments cause lower conservation scores when considered in calculation. With standing variation, conservation scores can be used off-the-shelf, i.e., as provided by UCSC Genome Browser and EBI Ensembl services. Another issue with human-derived data is that it is generated from the ancestral sequenceperspective, i.e., the human reference allele is not the ancestral allele. Thus, several annotations must be switched in order to match the human reference perspective. For example, a stop-loss variant in the human-derived data must be annotated as stop-gain before model training. Consequently, the annotation of start-loss and stop-gain variants is currently very limited. These and several other adaptations owing to the special origin of evolutionary data might lead to loss of information and introduce biases during training. Replacing human-derived data with standing variation overcomes these limitations, allowing to include new precalculated annotations into the training set.

Datasets based on standing variation also have some limitations. Rare and ultra-rare variants are used as an approximation for deleterious or pathogenic variants; however, many rare variants will not have a detrimental effect on fitness. While the proxy-deleterious set of CADD also has an unknown rate of mislabeled variants [15], its mislabeling is estimated to be 85%–95%—depending on the definition of functional constraint [66]. Estimation of the true proportion of deleterious variants among rare variants is also challenging and varies significantly [41]. Based on the results presented here and in our previous work, we see evidence that even substantial mislabeling in very large datasets and with simple or highly regularized model architectures can deal with substantial mislabeling and has limited impact on model performance.

Another issue arises from potential sequence errors in the case of ultra-rare variants. In general, the construction of databases of genomic variants is a complex process, where errors can occur and accumulate during each step including sequencing, mapping to the reference genome, and variant calling [67–69]. Extensive efforts have been made towards the reduction of overall error rates like improving variant calling using recent advancements in machine learning [70] or Telomere-to-Telomere sequencing of the genome [71]. Nonetheless, errors in databases of sequenced population data remain an important issue but might be compensated for by the sheer number of rare variants and probably have a limited impact on simple models.

A further drawback of the available variant data lies in the lack of ethnic diversity, which might negatively affect the allelic frequencies reported in datasets of genomic data. Out of 76,156 sequenced genomes in gnomAD v3.0, almost 52% are of European and 27% of African ancestry [22]. The new release of gnomAD v4.0 includes more than 400,000 exomes from the UK Biobank, where individuals of European ancestry account for over 94% of sequenced individuals [72]. It is known that, for example, African populations have higher genetic diversity, and populations in general differ in the composition of common and rare variants [73, 74]. Thus, it is possible that rare or singleton variants in widespread databases of genomic data are common polymorphisms of underrepresented populations. A solution here would be to use allele frequencies on a population level, assuming that a common benign variant in one population is benign in all populations. An initial analysis suggests that this could for example increase the number of proxy-benign by more than 40%.

The gnomAD v3.0 release used for this study excluded exome data to obtain an even coverage of sequence variants along the genome and across functional effects. However, it might be argued that a call set including exome capture data increases the coverage of high-impact functional effects and would improve model training, something that we did not explore here. We note though that CADD v1.6 outperforms most of the alternative models in coding regions, for example for synonymous and non-synonymous variant effects, where such a combination of exomes and genomes might give more weight to these molecular effects in training and thus improve the performance of models based on standing variation. The recently released gnomAD v4 release includes up to 730,947 exomes from various sources. Since the latest release available to us during the time of the study (v4.0) contained a known issue that affected allele frequencies [75], we did not consider it for our study.

A general issue for benchmarking model performance of variant prioritization methods is the availability of validation sets. To compare model performance, we utilized the NCBI ClinVar database, which, despite being broadly used, has several drawbacks. For one, like all available databases of pathogenic variants, it is biased towards the coding genome, thus containing an unproportionally small amount of noncoding variants, and even in the coding space, it is biased towards well-studied genes [3, 4, 76]. So, if an alternative model has superior performance in noncoding genomic regions, the power to detect it on this validation set is limited. Secondly, the benign variants contained in the ClinVar dataset can be regarded only as conditionally benign as many of them were initially candidate disease variants or were at least prioritized with a clinical analysis pipeline, and later labeled as benign or Variant of Uncertain Significance (VUS) after obtaining no confirmation of their pathogenicity. This, nevertheless, does not mean that these “benign variants” cannot be causative for any other genetic disease; i.e., “absence of evidence” does not necessarily mean “evidence of absence” [6]. To overcome this issue, many studies including CADD v1.6 and v1.7 use common variation with MAF > 1% or 5% from population studies as a benign set and contrast them with the pathogenic variation from ClinVar to validate results. However, since common variation is a part of some of the alternative training sets, we did not follow this approach to avoid inflated performance of models based on frequent variants.

The pervasive issue of validation sets is underlined by the comparison of alternative models and CADD v1.6 with the newly released CADD v1.7. It is trained on an augmented set of annotations, including state-of-the-art protein language model scores, regulatory variant effect predictions, Zoonomia species conservation measures, and additional noncoding scores. Nonetheless, the general comparison of, e.g., CADD v1.6 and CADD v1.7 on NCBI ClinVar data did not reveal significant performance gains, with their recall and precision scores being very close. However, the superiority of CADD v1.7 over the previous version has been shown on specialized datasets in its publication [34]. Most importantly, the comparison with CADD v1.7 highlights a potential of achieving similar or better performance from alternative labeled training data sets rather than only relying on updates and advances in model features, suggesting that the combination of both will produce even better models in the future.

## Conclusions

Here, we present several training sets based on standing variation from gnomAD v3.0, approximating variant deleteriousness with low or high allele frequency in the population. We show that using frequent standing variation as a proxy for benign and rare or singleton variation as a proxy for deleterious variants allows us to train state-of-the-art models for genome-wide variant prioritization. The proposed datasets have several advantages, like being significantly larger and potentially less biased than conventional datasets for deleteriousness scoring. Consequently, some of the alternative models outperform broadly used scores for variant prioritization, CADD v1.6 and v1.7, in predictions of pathogenic and benign variant classes from the NCBI ClinVar database.

Covering a broader range of rare annotations and containing recent evolutionary changes, large datasets of standing variation can be used either standalone or in combination with evolutionary data, for example human-derived variants or primate variation data, for various research purposes. Across various training data settings, we show that these alternative datasets can improve variant prioritization, providing better assessment of variant pathogenicity, especially also for non-coding effects. We make these training sets readily available for reproducing our results on the CADD v1.6 feature set, but also for annotating them with other feature sets. Future models derived from the variant sets provided will enable various basic research and clinical applications.

## Supplementary Information

Additional file 1. Supplementary Figures S1–S7 and Tables S1–S4.

## Data Availability

Annotated training data sets as well as model coefficients are available on Zenodo [77] at https://zenodo.org/records/13832126.

## Abbreviations

CADD: Combined Annotation Dependent Depletion
AUROC: Area Under Receiver Operating Characteristic
AUPRC: Area Under Precision Recall Curve
gnomAD: Genome Aggregation Database
MAF: Minor allele frequency
SNV: Single nucleotide variant
InDel: Insertion/deletion
